# New application of Diffusion Tensor Imaging in neurosurgery

**Published:** 2011-11-24

**Authors:** RE Rizea, AV Ciurea, G Onose, RM Gorgan, A Tascu, F Brehar

**Affiliations:** * Neurosurgery Department, “Bagdasar Arseni” Clinical Emergency Hospital, Bucharest, 10–12 Berceni Av., District 4, Romania; ** Neurorehabilitation Department, “Bagdasar Arseni” Clinical Emergency Hospital, Bucharest, 10–12 Berceni Av., District 4, Romania

**Keywords:** fractional anisotropy, mean diffusivity, tumor cell density

## Abstract

Diffusion tensor imaging is a MRI technique that enables the measurement of the diffusion of water in tissue in order to produce neural tract images. Advanced methods such as color coding and tractography (fiber tracking) have been used to investigate the directionality. The localization of tumors in relation to the white matter tracts (infiltration, deflection), has been one the most important initial applications. A non invasive technique for assessing tumor tissue characteristics, like tumor cell density, is required to assist preoperative surgical planning for malignant brain tumors and help better deﬁne the target for tumor biopsy, resulting in more accurate diagnosis and grading of malignant brain tumors. One possible source of this information is diffusion tensor imaging. Date studies have focused on its ability to delineate white matter ﬁber tracks by ﬁber tracking and to detect tumor inﬁltration around the tumor and normal white matter interface. Relationships between cell density and the two key values that diffusion tensor imaging provides, fractional anisotropy and mean diffusivity, still need to be investigated. Mean diffusivity has a good negative correlation and fractional anisotropy has a good positive correlation with tumor cell density within the tumor core. Similar correlation was observed between the Ki–67, on the one hand and fractional anisotropy and mean diffusivity, on the other hand. Thus, measurement of both fractional anisotropy and mean diffusivity within the tumor core has a potential to provide detailed information on tumor cell density within the tumor.

**Abbreviations:** DTI - diffusion tensor imaging, FA - fractional anisotropy, MD - mean diffusivity, WM - white matter

## Introduction

**Fig. 1:**
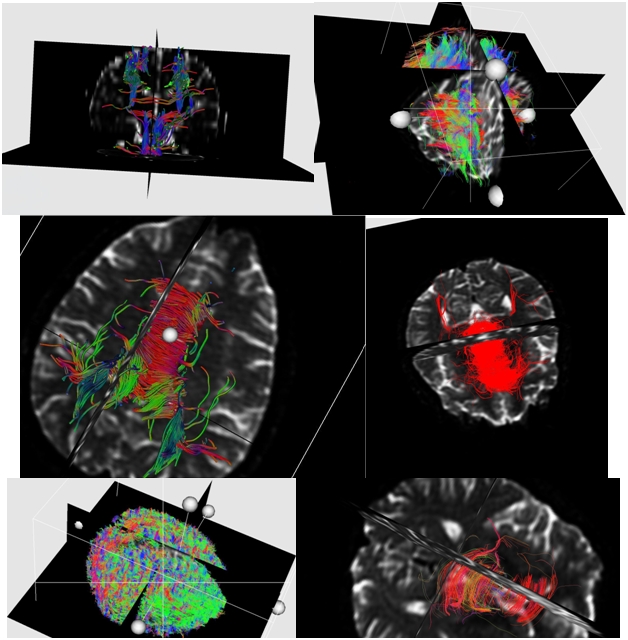
1 The first images of tractography from our research project. Fibers in red color are left–right. Fibers in green color are anterior–posterior. Fibers in blue color are superior–inferior

Diffusion tensor imaging (DTI) is a MRI technique that enables the measurement of the diffusion of water in tissue in order to produce neural tract images (**Fig. 1**). The idea of using diffusion data to produce images of neural tracts was first proposed by Aaron Filler & colleagues in March of 1991. Several months later (1992) the first DTI image showing neural tracts curving through the brain was produced.

Recent development of magnetic resonance imaging (MRI) has made it possible to delineate detailed anatomical and functional information of both normal and pathological brain tissues in a noninvasive manner. Among them, diffusion tensor MR imaging (DTI), has attracted great interest in that it can provide neural ﬁber information by use of the ﬁber–tracking technique and can also detect the presence of tumor cell inﬁltration into the normal white matter around the tumor boarder. The target in the treatment of malignant brain tumors is to maximize tumor resection volume, information on neural ﬁber preservation and tumor cell inﬁltration is critical for preoperative neurosurgical planning [**1**].

DTI data can be used to visualize the major white matter (WM) tracts of the brain [**2–4**]. DTI is an MR technique that can indirectly evaluate the integrity of WM by measuring water diffusion and its directionality in three dimensions [**5**]. DTI has been applied to differentiate edema from tumor, in patients with brain tumor for tumor characterization and to assess structural properties of the adjacent tracts [**6–11**]. Knowledge of the anatomical relationship between tumor and WM tracts could improve preoperative risk analysis and decrease the risk of WM tract injury during surgery.

Multiple studies investigated the relationships between fractional anisotropy (FA) values, mean diffusivity (MD) values and the histological tumor cell inﬁltration within the region of interest where the FA and MD were measured [**12–14**]. These studies have demonstrated that FA values tend to decrease in locations where the neural ﬁbers have been inﬁltrated by tumor cells, suggesting that relatively low FA values indicate tumor cell invasion into white matter, where neural ﬁbers reside.

The relationships between tumor cell density and FA or MD are still controversial, as both positive and negative correlation between these parameters has been reported [**13,15,16**]. Thus, the development of preoperative histological evaluation of tumors by noninvasive imaging techniques would greatly assist the preoperative diagnosis of the lesion and also preoperative surgical planning of the operation. For example, when a tumor biopsy of a glioma is required, it is extremely important that the target for biopsy is the location with the highest cell density, to allow accurate histological diagnosis.

## Methods

The principal application of DTI is in the imaging of white matter where the location, orientation, and anisotropy of the tracts can be measured. DTI allows clinicians to look at anisotropic diffusion in white-matter tracts, but it is limited in demonstrating spatial and directional anisotropy. Advanced methods such as color coding and tractography (fiber tracking) have been used to investigate the directionality. If diffusion gradients (i.e. magnetic field variations in the MRI magnet) are applied in at least 3 directions, 6 directions improve the accuracy that describes the 3-dimensional shape of diffusion.

The fiber direction is indicated by the tensor’s main eigenvector which can be color–coded, yielding a cartography of the tracts' position and direction: red for left–right, blue for superior–inferior, green for anterior–posterior.

Tractography potentially solves a problem for a neurosurgeon in terms of minimizing functional damage and determining the extent of diffuse infiltration of pathologic tissue to minimize residual tumor volume. In this way, tractography facilitates preoperative planning. Tractographic images may help to clarify whether a tumor is compressing, abutting, or infiltrating the contiguous white–matter tracts. DTI identify different tumor components, and to differentiate tumor invasion from normal brain tissue or edema.

DTI has demonstrated a potential in distinguishing gliomas and solitary metastasis in the brain parenchyma. Significantly higher MD, compared with levels in normal–appearing white matter, has been demonstrated in the peritumoral regions of both gliomas and metastases. Peritumoral MD of metastases and meningioma is significantly higher than that of gliomas.

The FA and MD measurement in region of interest can be selected anatomically by referring to the MR images of preoperative surgical planning and postoperative results in order to match the location from which the histological specimen can be obtained. FA and MD values were can be calculated using a computer with the image analyzing software like MedINRIA. After surgical resection, specimens can be ﬁxed in formalin and embedded with parafﬁn; hematoxylin and eosin-stained specimens were checked to determine the histological tumor type. Cell counting can be performed under a light microscope at standard magnification like ×200. A standard area for the tumor cell density can be established and can be used a program like ImageJ for counting. Also, Ki–67 labeled cells can be counted and the percentage of Ki–67 labeled cells can be calculated within the observed ﬁeld. [**17**]

The localization of tumors in relation to the white matter tracts (infiltration, deflection), has been one the most important initial applications. In surgical planning for some types of brain tumors, surgery is aided by knowing the proximity and relative position of tumor.

## Discussions and Results

Tractography combined with functional MRI may potentially help in preoperative planning of brain tumors by mapping areas of active infiltration. The recent development of DTI allows for direct examination of the brain microstructure.

In the surgery of patients with brain tumors, preservation of vital cerebral function is as important as maximizing tumor resection. The associated morbidity of aggressive resections can be significantly reduced by carefully preservation of vital cerebral function, and the quality of life of these patients will be largely improved.

In general, cerebral tumor may alter the adjacent WM in three different ways: by (1) displacing the WM tracts but with relative preservation of the fibers, (2) infiltrating the WM tracts, and (3) disrupting of the WM tracts. Intracranial tumors may involve both functional cortical gray and white matter tracts.

Preoperative characterization of tumor tissue using noninvasive imaging modalities is one of many necessary steps for establishing a successful treatment strategy against malignant brain tumors. It is widely accepted that tumor cell density correlates with tumor malignancy. In cases where a tumor biopsy is required, the target is preferentially chosen at the lesion with the highest tumor cell density, to achieve accurate diagnosis and grading of the disease. In cases of contrast agent–enhancing tumors, usually the target is set at the location with distinct enhancement on MRI, based on the known fact that the blood–brain barrier is disrupted within the lesion with higher malignancy, enabling contrast agents to enhance the area [**18**]. Perfusion weighted MR imaging (PWI), chemical shift imaging (CSI) and positron emission tomography (PET) have been proposed for retrieving information on biological characteristics of the tumor in a noninvasive fashion [**19,20**].

The FA and MD can be calculated and the results indicated an FA signiﬁcantly lower in the low-grade gliomas than the malignant lymphomas. [**17**] This observation is similar to the conclusion of Toh et al. (2008) [**21**]. The average indicated MD signiﬁcantly higher in low–grade gliomas and also in high-grade gliomas compared to malignant lymphomas. These ﬁndings suggest that MD is more sensitive than FA in distinguishing gliomas from malignant lymphomas.

Tumor density of the tumor core shows positive correlation with FA but negative correlation with MD. These ﬁndings are compatible with those previously reported by Beppu et al. (2003, 2005) [**15,16**] but contradict those of Stadlbauer et al. (2006) [**13**]. Although a direct comparison of FA and MD was not performed in the study by Stadlbauer et al., they showed that tumor cell density has negative correlation with FA and positive correlation with MD [**13**]. Thus it seems fair to speculate that the data in the study of Stadlbauer et al. also shows an inverse relationship between FA and MD values [**13**]. In addition, an inverse relationship between FA and MD has also been reported for multiple sclerosis [**22**]. One possible explanation for FA and MD show inverse correlation is the presence of tumor cells within the tissue prevents water diffusing in a single direction, which would show both high FA and MD values.

Previous studies have focused the use of diffusion tensor MR imaging (DTI) for ﬁber–tracking [**23–30**] and evaluating tumor inﬁltration near the tumor boarder [**12–14**]. These assessments are crucial for achieving maximum tumor resection of malignant brain tumors.

Ki–67 labeling index is positively correlated with FA and negatively correlated with MD. Tumor cell density at the core of the tumor and Ki–67 labeling index show good positive correlation, suggesting that tumor cell density and Ki–67 labeling index have diagnostic value in malignant brain tumors. [**17**].

As FA and MD (in combination) seem to represent the histology of these tumors, in particular of tumor cell density. It is expected that locations with higher FA and lower MD will have higher tumor cell density, as well as have a relatively high Ki–67 labeling index, indicating higher malignant potential. It should be noted that higher FA values could also implicate the contamination of high FA resulting from intact neural ﬁbers. [**17**]

## Conclusions

As a conclusion, data from multiple studies of DTI suggest to represent tumor tissue characteristics in a noninvasive fashion. When the enhancement by contrast agent is vague, other indicators are necessary to assess tumor cell density within the tumor. The use of diffusion tensor MR imaging (DTI), however, for this purpose still requires investigation.

**Acknowledgments** This investigation was supported by a Grant for Scientiﬁc Research from the Ministry of Education and Science of Romania (subject numbers: 42–149/2008).
